# The Onset of In-Vivo Dehydration in Gas -Based Intraperitoneal Hyperthermia and Its Cytotoxic Effects on Colon Cancer Cells

**DOI:** 10.3389/fonc.2022.927714

**Published:** 2022-06-29

**Authors:** Agata Diakun, Tanja Khosrawipour, Agata Mikolajczyk-Martinez, Jakub Nicpoń, Zdzisław Kiełbowicz, Przemysław Prządka, Bartłomiej Liszka, Wojciech Kielan, Kacper Zielinski, Pawel Migdal, Hien Lau, Shiri Li, Veria Khosrawipour

**Affiliations:** ^1^ 2nd Department of General Surgery and Surgical Oncology, Wroclaw Medical University, Wroclaw, Poland; ^2^ Department of Surgery (A), University-Hospital Düsseldorf, Düsseldorf, Germany; ^3^ Medical faculty, Heinrich-Heine University, Düsseldorf, Germany; ^4^ Department of Biochemistry and Molecular Biology, Faculty of Veterinary Sciences, Wroclaw University of Environmental and Life Sciences, Wroclaw, Poland; ^5^ Department of Surgery, Faculty of Veterinary Sciences, Wroclaw University of Environmental and Life Sciences, Wroclaw, Poland; ^6^ Department of Anesthesiology, Wroclaw Medical University, Wroclaw, Poland; ^7^ Department of Environment, Hygiene and Animal Welfare, University of Environmental and Life Sciences, Wroclaw, Poland; ^8^ Department of Surgery, University of California, Irvine, Irvine, CA, United States; ^9^ Division of Colon and Rectal Surgery, Department of Surgery, New York Presbyterian Hospital- Weill Cornell College of Medicine, New York, NY, United States; ^10^ Department of Surgery, Petrus-Hospital Wuppertal, Wuppertal, Germany

**Keywords:** dehydration, colorectal cancer, peritoneal metastasis (PM), hyperthermia, electron microscopy

## Abstract

**Background:**

Peritoneal metastasis (PM) is an ongoing challenge in surgical oncology. Current therapeutic options, including intravenous and intraperitoneal (i.p.) chemotherapies display limited clinical efficacy, resulting in an overall poor prognosis in affected patients. Combined hyperthermia and dehydration induced by a high-flow, gas-based i.p. hyperthermic procedure could be a novel approach in PM treatment. Our study is the first to evaluate the therapeutic potential of i.p. dehydration, hyperthermia, as well as the combination of both mechanisms in an *in-vivo* setting.

**Methods:**

For this study, three swine were subjected to diagnostic laparoscopy under a high-flow air stream at 48°, 49° and 50°Celsius (C). Hygrometry of the in- and outflow airstream was measured to calculate surface evaporation and i.p. dehydration. To analyze the effects of this concept, *in vitro* colon cancer cells (HT-29) were treated with hyperthermia and dehydration. Cytotoxicity and cell viability were measured at different time intervals. Additionally, structural changes of dehydrated cells were analyzed using scanning electron microscopy.

**Results:**

According to our results, both dehydration and hyperthermia were cytotoxic to HT-29 cells. However, while dehydration reduced cell viability, hyperthermia did not. However, dehydration effects on cell viability were significantly increased when combined with hyperthermia (p<0.01).

**Conclusions:**

Changes to the physiological milieu of the peritoneal cavity could significantly reduce PM. Therefore, limited dehydration of the abdominal cavity might be a feasible, additional tool in PM treatment. Further studies are required to investigate dehydration effects and their applicability in PM management.

## Introduction

While peritoneal metastasis (PM) is a common manifestation of advanced gastrointestinal and gynecological cancers, it remains a challenge to surgical oncology with an overall poor prognosis. Statistically, affected patients do not survive the first year after diagnosis due to rapid tumor progression in the abdominal cavity and subsequent complications (1, 2). In recent years, various attempts have been made to develop new strategies for PM management. Despite these efforts, there have not been any significant scientific developments that ensure long-term clinical improvement.

In fact, one of the main shortcomings observed with current therapies include limited therapeutic effects observed with systemic chemotherapy. This can be attributed to systemic drug loss and subtherapeutic drug concentrations in the peritoneum (3, 4). As a result, locoregional concepts e.g. intraperitoneal (i.p.) chemotherapy have been introduced to further improve local drug availability. However, later studies uncovered that i.p. chemotherapies also display limited clinical efficacy (5). Currently, the combination of cytoreductive surgery (CRS) with fluid based hyperthermic intraperitoneal chemotherapy (HIPEC), offers hope for a curative outcome in patients with limited disease. CRS and HIPEC is gaining more popularity in the field of surgery, as clinical trials describe advantageous results in highly selective patients (6, 7). Despite this encouraging data, it is important to note that only patients suffering from limited diseases and without visible peritoneal seeding following CRS are considered to benefit from this therapeutic option (8, 9). For patients with extensive PM who do not qualify for CRS and HIPEC, the option of pressurized intraperitoneal aerosol chemotherapy (PIPAC) has been introduced to the clinical setting (10, 11). In PIPAC, a fluid chemosolution is aerosolized and injected into the abdominal cavity covering the abdominal compartment. In fact, PIPAC has shown some positive results (12, 13) and several attempts have been made to improve (14, 15) and extend (16) its application. Nevertheless, the use of PIPAC in the clinical setting does not seem to fundamentally change patient outcome.

Many patients without distant metastasis often display the clinical picture of metastatic seeding in the peritoneal cavity. PM cells are often either confined to the peritoneum or minimally infiltrate local and peritoneal tissues (17, 18). It is suggested that peritoneal seeding is enhanced due to facilitated movement of floating cancer cells within the peritoneum (19, 20). The presence of disseminated cancer cells has been associated with a higher recurrence rate and a poorer survival (21). Using the peritoneal milieu as a favorable habitat for growth, adherent cancer cells can further infiltrate and permeate into tissues (22, 23). Additionally, the glucose and protein-rich cavitary fluid can further enhance this effect. In fact, the abdominal cavity offers optimal growth potential for local malignant tumor cells. Consequently, we assume that changing the biology of this compartment could lead the way to optimized therapies. Thus, it is necessary to investigate whether changing the biological properties of the peritoneum could be a feasible option to delay or even halt PM progression.

The application of dehydration combined with hyperthermia could be an innovative approach to alter the biological properties of this compartment. Malignant tumor cell progression could be terminated in a less hospitable environment. Our study is in strong contrast to previous attempts, which all included the use of chemotherapeutic applications. By means of this study, we aim to calculate the extent of evaporation and dehydration that may occur during laparoscopy. Finally, using an *in-vivo* swine model, we examine whether dehydration can be achieved by the proposed laparoscopic system as well as analyze the sensitivity of colon cancer cells to hyperthermia and dehydration at different levels of exposure.

## Material and Methods

### *In-Vivo* Swine Model

**Figure 1:**
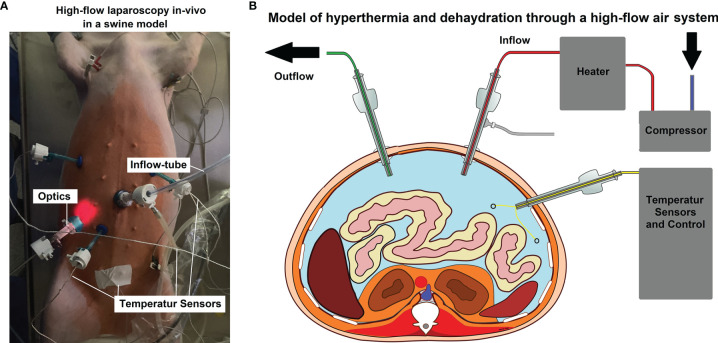
**(A)** *In-vivo* high-flow laparoscopy model to evaluate dehydration and hyperthermic airflow effects. **(B)** Model of the laparoscopic setting. Two trocars are used as in- and outflow while other trocars are placed for optics and temperature sensors.

For the *in-vivo* part of this study, three 65-day-old swine (Polish white flod) were used. The swine received diagnostic laparoscopy without any additionally surgical intervention. Laparoscopy was performed under a high-flow air stream of 15 liters per minute (l/min). The swine used were part of a larger study on hyperthermia and dehydration. The inflowing air temperature for the swine were 48°, 49 and 50°Celsius (C), respectively. Intracavitary temperatures were monitored to detect the onset of critical intraabdominal heating. All animals received humane care in compliance with the Guide for the Care and Use of Laboratory Animals as published by the National Institutes of Health. The swine were premedicated with an intramuscular injection of midazolam (0.3 mg/kg, WZF Polfa S.A., Poland), medetomidine (0.02 mg/kg, Cepetor 1 mg/ml, CP-Pharma Handelsgesellschaft, Germany) and ketamine (9 mg/kg, Ketamina 100 mg/ml, Biowet Puławy sp. z o.o., Poland) mixture. Analgesia was performed with Propofol at 1mg/kg. Swine were intubated and further anesthesia was continued with isoflurane 1%. Additional analgesia was provided with fentanyl 2µg/kg and crystalloid fluid at 0.2 - 0.3 µg/kg/min. The swine were placed in a supine position. An infra-umbilical mini laparotomy was performed and another one at about 8 cm distance to the first one. A 10 mm trocar (Kii^®^Balloon Blunt Tip System, Applied Medical, Rancho Santa Margarita, CA, USA) was inserted through the infra-umbilical trocar while multiple 5 mm trocars were placed at the other sites (**Figure 1A**) after insufflation. The abdominal cavity was insufflated with filtered room air *via* a tube entering the central 10 mm trocar. An initial diagnostic check-up was conducted *via* laparoscopic imaging using a 5 mm camera system (Karl Storz 5mm/30° Laparoscope/Tuttlingen, Germany) through a 5 mm trocar. After visual confirmation and placement of multiple temperature sensors (**Figure 1B**), the high-flow air stream was turned on at 15 l/min for a total of 45 minutes. Humidity levels at the in- and outflow was measured.

### Creating an Operative Model for Laparoscopic Dehydration of the Abdominal Cavity

The operative model for dehydration assumes a 4 Liter (*V_B_* = 4 L) abdominal capnoperitoneum, which corresponds to an average sized capnoperitoneum observed during laparoscopy. Humidity levels of in- and outflowing air were measured. Based on the mean humidity difference, the amount of removed water from the abdominal cavity was calculated. The saturation pressure of room air is 7297 Pascal and the maximum density of water vapor at 40°C is 0.051 kg/m³. At a constant flow of 15 l/min, a model for a time dependent removal rate was calculated.

### Cell Cultures

The human colorectal cancer cell lines HT-29 were purchased from the CLS (Cell Lines Service GmbH, Eppelheim, Germany). Cells were then grown in Dulbecco’s modified Eagle’s medium (DMEM - high glucose, Sigma-Aldrich, Poznan, Poland). The following supplements were added to the medium: 10% heat-inactivated fetal bovine serum (FBS, Gibco, Thermo Fisher Scientific, Poland), 2 mmol/L glutamine, 100 IU/mL penicillin, and 100 μg/mL streptomycin (Sigma-Aldrich). Cells were then incubated in a humidified 5% CO_2_ incubator (NuAire CO_2_ Incubator, Biogenet, Warszawa, Poland) at 37°C. Afterwards, 1.4 x 10^5^ cells per well were seeded in 24-well plates (TC Plate 24 Well, Standard, F, Sarstedt AG & Co. KG, Germany) and incubated for 48 hours. Cells subjected to electron microscopy (EM) analysis were seeded on sterile glasscovers.

### *In Vitro* Cell Dehydration

Cells were divided into three main groups and exposed to 37°, 45° and 48°C for 30 minutes. Each group was separately exposed to different durations of dehydration (0, 5, 10, 20, 25 and 30 minutes) (at 85% percent humidity) in the cell incubator. After 0 and 20 minutes of dehydration, cells that were seeded on sterile glasscovers were subjected to cellular analyses using scanning electron microscopy (SEM).

### Cytotoxicity Assay

To obtain maximum LDH activity, cell lysis reagent was added into each well following exposure to hyperthermia and dehydration and incubated at 37°C for 45 minutes. Spontaneous LDH activity was assessed for each group. Wells were incubated in darkness and at room temperature for 30 minutes, then a stop reagent solution was added and absorbance was measured at 490nm on a microplate reader (Tecan, Basel, Switzerland). Percentage of cytotoxicity was calculated using the following formula:

% cytotoxicity = (compound-treated LDH activity – spontaneous LDH activity) ÷ (Maximum LDH activity – Spontaneous LDH activity) x 100%.

### MTS-*Testing* on *Cell Viability*

Cell proliferation was determined *via* colorimetric CellTiter 96^®^ AQueous One Solution assay (Promega, Poland) 24 hours after hyperthermia and dehydration exposure. The test was performed with our modifications according to the manufacturer’s instructions. Briefly, medium was removed from each well and replaced by 0.3 mL of fresh DMEM. Next, following one hour of incubation at 37°C at 5% CO_2_, an MTS-based reagent was added to each well and absorbance at 490nm was measured using a microplate reader (Tecan, Basel, Switzerland). The percentage of proliferation was regulated for all groups using untreated cells as control.

### Electron Microscopy to Determine Cell Structure and Single Cell Dehydration

The HT-29 control groups and dehydrated cells (20min) were subjected to cryogenic scanning electron microscopy (cryoSEM) analysis. The probes were washed with Dulbecco’s phosphate buffered saline (DPBS, Sigma-Aldrich) and fixated in 2.5% glutaraldehyde solution (Sigma-Aldrich). After fixing the samples, they were washed in PBS, rinsed in ultrapure (sterilized through 0.1µm filter) deionized water, and finally mounted on cryo-shuttle using OT/colloid graphite mixture and plunged in liquid nitrogen. Then, frozen specimen were quickly transferred to a cryo-preparation chamber (Cryo Quorum PP3010T) and sputtered with a conductive layer of platinum at 140°C. Next, specimen were transferred to the microscopy chamber. At each point of transfer, the same temperature of -140°C (Auriga60, Zeiss) was maintained. Samples were ultimately observed at 2kV of acceleration voltage using In Lens and SE2 secondary electron detector.

### Graphic *D*esign

For the graphics provided, multiple graphic programs were used. These programs included Inkscape 1.0.1,2020, GNU, USA and programs provided by Windows office 2019, Microsoft.

### Statistical Analysis

All experiments were executed at least three times. Each well was considered as a single value, according to the classified subgroups. The student t-test was used to compare independent groups. Probability (p) values were considered as follows: *=p<0.05, **=p<0.01, and #=p>0.05, with p-value <0.05 considered to be statistically significant. Data are presented as the mean standard deviation unless otherwise indicated.

## Results

### Extent of Laparoscopic Intraabdominal Dehydration and Transport Capacity

**Figure 2:**
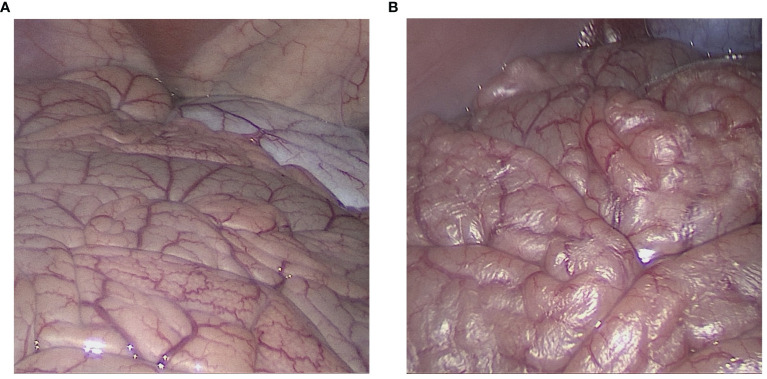
Laparoscopic view of the intraabdominal cavity during high-flow air-based hyperthermia. **(A)** Start of the laparoscopy. The figure displays normal small intestinal tissue as is expected during laparoscopy. **(B)** Laparoscopic view of the intraabdominal cavity after 30 minutes into the procedure. Peritoneal dehydration “drying” is visible in the exposed area. The peritoneal surface appears to “peel-off” and light reflection on the peritoneal surface increases.

**Figure 3:**
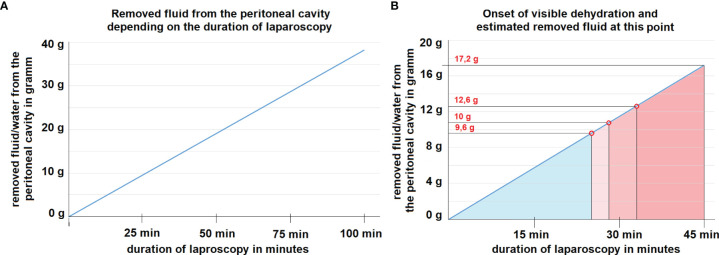
**(A)** Mathematical model for the removal of fluid/water from the abdominal cavity at 15 liter/minute flow rate assuming an inflow air humidity of 50%. The correlation is linear. **(B)** Mathematical model of dehydration at 15 liter/minute flow rate assuming an inflow air humidity of 50%. At the marked red points, dehydration of tissue is visible during laparoscopy. The estimated amount of removed water per time is indicated at each time point.

Under the presented constellation at 15 l/min flow, outflow humidity levels reached > 95% within the first 5 minutes and stabilized at around 99%. The mean inflow humidity level was 51%. The intraperitoneal temperature remained stable and did not exceed 40°C. Macroscopically visible dehydration set in after 25, 28 and 33 minutes within the procedure (**Figures 2A, B**). The calculation of the maximum transport capacity uncovered that under the presented constellation of high flow *via* the laparoscopic approach, a mean amount of 0.382 grams per minute of water was removed from the peritoneum (**Figure 3A**). This approximated to a total of 17.2 grams of water in 45 minutes (**Figure 3B**). Thus, dehydration set in after the removal of circa 10.7 ± 1.6 g of water from the abdominal cavity.

### *In-Vitro* Cytotoxicity of Dehydration and Hyperthermia

**Figure 4:**
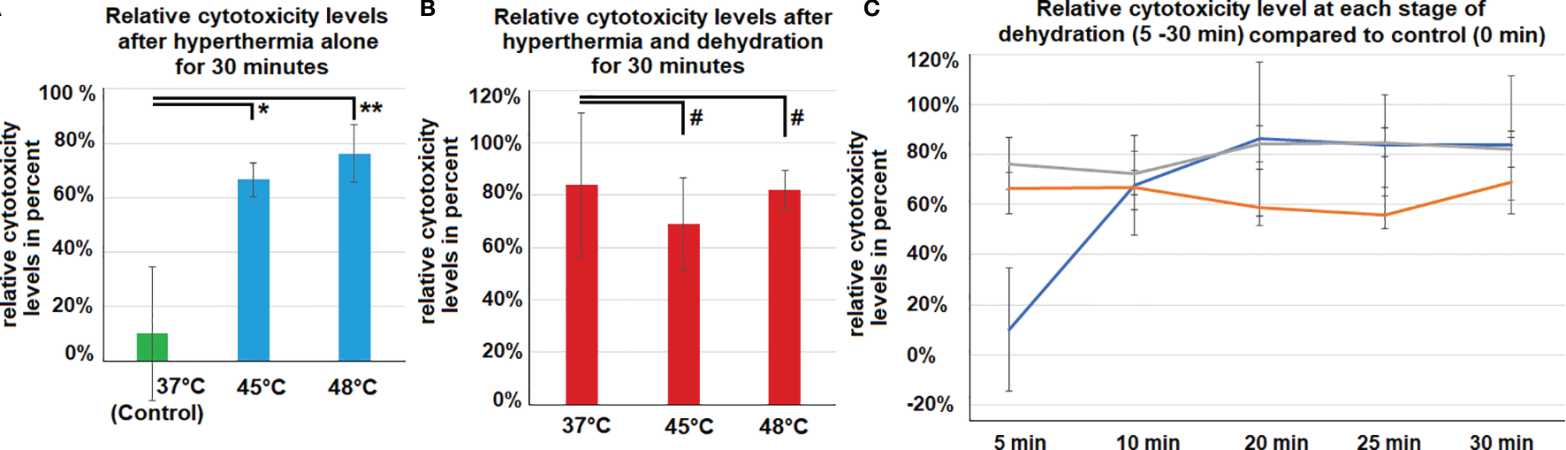
**(A)** *In vitro* cytotoxicity on colon cancer cells after 30 minutes of hyperthermia exposure (without dehydration) at 45°C and 48°C. Control cells were only exposed to the physiological temperature of 37°C. **(B)** *In vitro* cytotoxicity on colon cancer cells after 30 minutes of hyperthermia exposure and dehydration at 37°, 45°C and 48°C. **(C)** Cytotoxicity compared to control cells. Levels of cytotoxicity were measured at different exposure times to dehydration and hyperthermia (5, 10, 20, 25 and 30 minutes). Significance levels: #=p>0.05, *=p<0.05, **= p<0.001.

Cytotoxicity levels were significantly increased following hyperthermia at 45°C (p<0.05) and 48°C (p<0.001) compared to controls at 37°C (**Figure 4A**). Moreover, the combination of hyperthermia and dehydration showed similarly high cytotoxicity levels (**Figure 4B**). After 10 minutes of dehydration, cytotoxicity levels were high for all temperature groups and remained at this elevated level. However, in cells kept at 37°C and only exposed to dehydration, cytotoxicity increased to elevated levels after 10 minutes (**Figure 4C**).

### Viability of HT-29 Cells Following Dehydration and Hyperthermia

**Figure 5:**
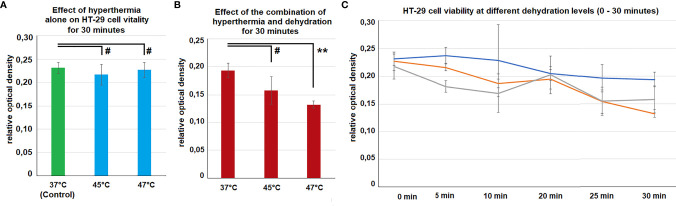
**(A)** *In vitro* cell-viability of colon cancer cells after 30 minutes of hyperthermia (without dehydration) at 45°C and 48°C. Control cells were only exposed to the physiological temperature of 37°C. **(B)** *In vitro* cell-viability of colon cancer cells after 30 minutes of hyperthermia and dehydration at 37°, 45°C and 48°C. **(C)** Course of cell viability following different exposure times of dehydration and hyperthermia 0 (Control), 5, 10, 20, 25 and 30 minutes. Significance levels: #=p>0.05,**= p<0.01.

The viability of HT-29 cells was not affected by hyperthermia at 45°C and 48°C compared to controls at 37°C (**Figure 5A**). However, the combination of hyperthermia at 48°C and dehydration of 30 minutes resulted in significantly decreased viability levels (p<0.01) (**Figure 5B**). Dehydration of cells at 37°C resulted in declined viability with time (0 - 30 minutes). However, this effect seemed to be further enhanced when hyperthermia was added at 45°C and 48°C (**Figure 5C**).

### Electron Microscopic Analysis on Dehydrated HT-29 Cells

**Figure 6:**
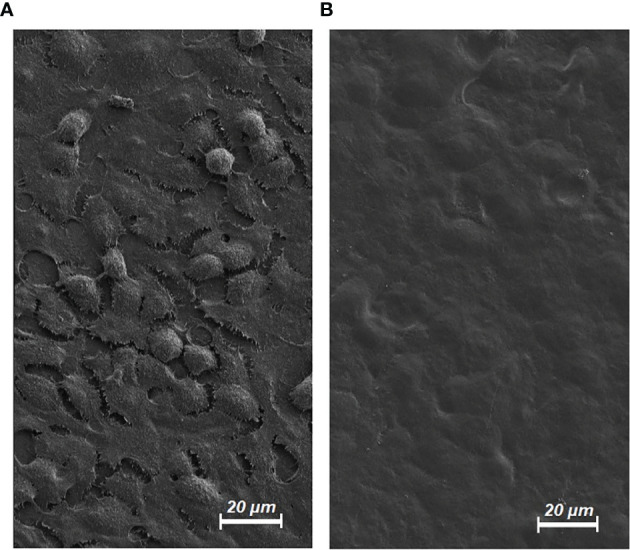
Electron microscopy (EM) of HT-29 cells. **(A)** Untreated HT-29 Cells in EM (control/magnification level 1000X). **(B)** HT-29 Cells in EM following dehydration and hyperthermia for 20 minutes (magnification level 1000X).

Cryo-SEM was used to study cell morphology following partial dehydration. Structural analyses of the extracellular cell surface revealed morphological changes which progressed with increased extent of dehydration. Control cells were more distinguishable and separable from each other. Some had a round shape and podocytes were visible for most cells (**Figure 6A**). Their dehydrated counterparts had blurred margins, and the cells appeared hollow and flattened (**Figure 6B**). Cell boundaries were more difficult to identify. Podocyte structures of cells had become visually unidentifiable. EM analyses concluded that dehydration significantly affected the structural integrity of HT-29 cells.

## Discussion

The concept of a therapeutic pneumoperitoneum was inspired by Italian physician Carlo Forlanini (1847 - 1918), whose pioneering innovations helped to manage extensive pulmonary tuberculosis following decades of stagnating therapeutic options. In the early 20^th^ century, he developed the idea of an artificial pneumothorax for patients suffering from pulmonary tuberculosis. This innovative concept benefited severely ill patients, and Forlanini was later nominated for the Nobel prize for medicine for his discovery (24). Based on the consideration that biophysical changes of the thorax and a less hospitable environment for tuberculosis bacteria might have been the reason for the success of this unconventional approach, we theorize that our model of cell dehydration could have similar promising effects on PM. In patients with cancer diseases, the metastatic process in the peritoneal cavity is a critical factor for survival (25). Using physical principles in innovative treatments of PM and other surface malignancies seems promising and has been attempted several times (26–29). Some of these novel treatment options have been extensively tested, including the application of irradiation (30–32), high-intensity ultrasound (33, 34) and nanoparticles (16). The current management of PM displays significant shortcomings due to the aggressive growth behavior displayed by PM, which further limits effects of i.p. chemotherapy even when locally applied during HIPEC or PIPAC.

Cancer cells within the abdominal cavity usually originate from either gastrointestinal or gynecological cells. Therefore, they are habituated to a fluid-surrounding environment. This aspect remains unchanged, regardless of mutations in the cancer cell genome (35). Consequently, changing the basic biology of the abdominal cavity might interfere with any cancerous progression in that compartment. Dehydrating the internal cavity accounts for one of these basic biological changes in the human body. With this study, we were able to demonstrate that HT-29 cells subjected to dehydration show reduced viability rates, especially when combined with hyperthermia. Our model has thoroughly demonstrated that dehydration can be implemented in a laparoscopic model established within a reasonable timeframe of less than 45 minutes. With our work, we have outlined a basic conceptual comprehension of the technical and physical aspects as well as applicational limitations of dehydration. This approach needs to be further investigated to evaluate the potential of i.p. dehydration in the management of advanced PM. Dehydration with hyperthermia as a combined application or possibly in addition to chemotherapy, could be a realistic tool for changing the i.p. environment to slow down or even terminate PM progression. Significant reduction of HT-29 cell viability and significantly increased cytotoxicity has been observed following dehydration combined with hyperthermia. Applicational, biological and technical features of dehydration within the peritoneal cavity have been demonstrated and must be further examined and documented. While this new concept operates without any pharmacological agents, it can possibly be combined with some of the current concepts of i.p. chemotherapy (36, 37).

With regards to limitations in this study, we must point out that PM can originate from different cancer entities, including a variety of gastrointestinal or gynecological tumors, and thus may show differing reactions to dehydration. Furthermore, there is still a limited understanding and knowledge on potential local and systemic effects of this novel concept. Also, the potential hemodynamic effects of dehydration and hyperthermia must be further evaluated. In the future, clinical studies in selected and qualified research centers are required to further assess this concept and allow for a more in-depth evaluation of its applicability.

## Data Availability Statement

The original contributions presented in the study are included in the article/supplementary material. Further inquiries can be directed to the corresponding author.

## Ethics Statement

The animal study was reviewed and approved by Wroclaw university of life and environmental sciences.

## Author Contributions

AD: Study design, laboratory analysis, data acquisition, editing. TK: Study design, conception of the study and manuscript drafting. AM-M: Study design, laboratory analysis, data acquisition. JN: Study design, laboratory analysis, data acquisition. ZK: Manuscript drafting and critical revision for important intellectual content of the manuscript. PP: laboratory analysis, data acquisition. BL: Study design, laboratory analysis. WK: Drafting and critical revision for important intellectual content of the manuscript. KZ: laboratory analysis, data acquisition, PM: laboratory analysis, data acquisition. HL: Drafting and critical revision for important intellectual content of the manuscript. SL: Drafting and critical revision for important intellectual content of the manuscript. VK: Supervision on study design, laboratory analyses, conception of the study and manuscript drafting. All authors contributed to the article and approved the submitted version.

## Funding

This study was funded by institutional funds of the Department of Biochemistry and Molecular Biology, Faculty of Veterinary Sciences, Wroclaw University of Environmental and Life Sciences, Wroclaw, Poland and the Department of Surgery, Petrus-Hospital Wuppertal, Wuppertal, Germany.

## Conflict of Interest

The authors declare that the research was conducted in the absence of any commercial or financial relationships that could be construed as a potential conflict of interest.

## Publisher’s Note

All claims expressed in this article are solely those of the authors and do not necessarily represent those of their affiliated organizations, or those of the publisher, the editors and the reviewers. Any product that may be evaluated in this article, or claim that may be made by its manufacturer, is not guaranteed or endorsed by the publisher.
